# The effect of adjusting settings within a Computer-Assisted Sperm Analysis (CASA) system on bovine sperm motility and morphology results

**DOI:** 10.1590/1984-3143-AR2021-0077

**Published:** 2022-02-04

**Authors:** Ciara O’Meara, Emilie Henrotte, Kasia Kupisiewicz, Catherine Latour, Marleen Broekhuijse, Agnes Camus, Lucie Gavin-Plagne, Eli Sellem

**Affiliations:** 1 National Cattle Breeding Centre, Naas, County Kildare, Ireland; 2 Production and Distribution Direction, Awé Groupe, Inovéo, Ciney, Belgium; 3 Research and Development Department, Viking Genetics, Assentoft, Denmark; 4 Innovation Department, Coöperatie Rundvee Verbetering B.V., Arnhem, The Netherlands; 5 Innovation, Science et Technologies, IMV Technologies, L’Aigle, France; 6 Innovations et Developements, ALLICE, Paris, France

**Keywords:** bovine semen, sperm functionality, settings standardization

## Abstract

Semen motility is the most widely recognized semen quality parameter used by Artificial Insemination (AI) centers. With the increasing worldwide export of semen between AI centers there is an increasing need for standardized motility assessment methods. Computer-Assisted Sperm Analysis (CASA) technology is thought to provide an objective motility evaluation; however, results can still vary between laboratories. The aim of present study was to verify the impact of different setting values of the CASA IVOS II on motility, concentration, and morphology of bovine semen samples frozen in an extender with or without egg yolk and then decide on optimal settings for a further validation step across AI centers. Semen straws from 30 different bulls were analyzed using IVOS II with twelve modified settings. No significant changes were observed in semen concentration, percentage of motile sperm or kinetic results for either extender type. However, increasing settings for both STR and VAP progressive (%) from Low, Medium, and High cut-off values significantly (p<0.05) reduced the percentage of detected progressive spermatozoa, in egg yolk extender from 49.5±15.2, 37.2±11.9 to 11.9±5.3%, and in clear extender from 51.9±9.1, 35.8±7.3 to 10.0±2.4%, respectively. In clear extender only, the modification of droplet proximal head length significantly affected the detection of normal sperm percentages (88.0± 4.7 to 95.0±0.6 and 96.0±0.6%) and of the percentage of detected proximal droplets (12.2±4.7, 2.5±2.7 to 0.6±0.2%) for Low, Medium and High values respectively (p<0.05). The identification of sensitivity within the CASA system to changes in set parameters then led to the determination of an optimal IVOS II setting. The existing variability among centers for these phenotypes was reduced when the standardized settings were applied across different CASA units. The results clearly show the importance of applied settings for the final CASA results and emphasize the need for standardized settings to obtain comparable data.

## Introduction

An important element in current artificial insemination (AI) practice is accurate and precise semen quality assessment. Many laboratories around the world analyze characteristics of semen quantity (including volume, sperm concentration, color, density, and viscosity) and more particularly sperm cell quality (including total and progressive motility (Rossitto et al., 2019), morphological abnormalities (Amann et al., 2000), mitopotential (Bucak et al., 2015), oxidation sensitivity (Partyka et al., 2011) and DNA fragmentation (Castro et al., 2018)). Several studies have been conducted in humans (Agarwal and Said, 2003; Keel, 2004), pigs (Broekhuijse et al., 2012), and bulls (Sellem et al., 2015; Christensen et al., 2011) to examine the relationship between such *in vitro* laboratory standards of fertile semen and their relationship with *in vivo* field fertility. All these analyses serve multiple purposes; however, their ultimate goal is to ensure the successful packaging of a straw with fertile spermatozoa that meets semen laboratory standards.

The standardization of assessment methods is becoming more and more necessary with the increasing worldwide export of semen straws and communication between animal breeding centers (Walczak-Jedrzejowska et al., 2013; Mallidis et al., 2012; Lu et al., 2010; Toft et al., 2005; Keel, 2004; Brazil et al., 2004; Keel et al., 2002). Standardization requires a large amount of organization and involves several laboratories working on the same samples. In some cases, the intra-laboratory variability of the assessments could be higher than the biological variability that truly exists among samples (Keel, 2004). Indeed, Brazil et al. (2004) identified large variation coefficients intra/inter laboratories in the range of 23 to 73% for the sperm concentration; 9 to 37% for the sperm motility; 25 to 87% for the morphological abnormalities assessment.

Strategies are in place in human clinical andrology, where The World Health Organization (WHO) recommends the use of an external laboratory to benchmark all technicians against the mean data for each parameter, aiming to reduce technical variability among technicians within laboratories (Walczak-Jedrzejowska et al., 2013; Mallidis et al., 2012).

Worldwide, the most commonly assessed semen quality parameter is motility. For years, the standard method in labs was via a subjective microscopic assessment (Mallidis et al., 2012). The WHO recommends subjective assessment by trained andrologists as the gold standard in human semen evaluation (WHO, 2010). Although this technique is accepted worldwide, it is highly dependent on training and laboratory proficiency testing, making it largely operator-dependent. To overcome this problem, objective technologies have emerged such as Computer Assisted Sperm Analysis (CASA) systems, that are used in the daily routine of semen processing. CASA is a system combining specific hardware and software design, a high-resolution camera, and a microscope that enables density and pixel recognition for computer-assisted evaluation of semen concentration, motility, velocity, and morphology (Amann and Waberski, 2014). However, even with computer systems, many studies have found variation within operators, machines of the same brand, and between different models (Verstegen et al., 2002; Hansen et al., 2006; Brito, 2010).

Hence, a European group of semen production centers and research labs were created in 2018 as part of the Annual EU AI Vets Congress QualiVets subgroup (knowledge exchange platform on all relevant subjects concerning AI station management, semen collection, evaluation of semen quality, semen dilution, semen storage, and semen transportation). This group brought together CRV (Arnhem, The Netherlands), Viking Genetics (Assentoft, Denmark), The National Cattle Breeding Centre / Ireland Genetics (NCBC, Kildare, Ireland), AWE group (Inovéo-Walloon breeding association, Ciney, Belgium), Allice (Paris, France) and IMV Technologies (L’Aigle, France) to develop a standardized international protocol for the phenotypic assessment of several semen quality parameters. Many factors need to be standardized, such as sample preparation, operational procedures, and analyses.

The objective of the present study, therefore, was to verify the impact of different settings of the CASA IVOS II (Hamilton Thorne Ltd, Beverly, USA) on motility, concentration, and morphology of bovine semen samples frozen in an extender with or without egg yolk. Following results, the group aimed to propose common, optimized, and standardized settings for CASA assessment of bovine samples according to the extender type.

## Materials and methods

### Sample collection and processing

Thirty semen straws from each of the following mature breeds: Belgian blue (N=20), Holstein-Friesian (N=6), Aberdeen Angus (N=1), Hereford (N=1), Limousin (N=1), and Simental (N=1) bulls were collected at either NCBC (Ireland) or AWE group (Belgium), using an artificial vagina. After collection semen quality parameters (ejaculate volume, sperm cell concentration, total motility, and progressive motility) were evaluated and ejaculates fulfilling AI center quality standards were processed as done previously in NCBC (Murphy et al., 2018) and AWE (internal standard ISO9001).

Approved ejaculates were fully extended in egg yolk media using either Optidyl (N = 10) or BullXcell (N = 10) or phospholipid-based media (N=10) OptiXcell (all extenders from IMV Technologies, L’Aigle, France) to achieve a final concentration between 15 and 25 x 10^6^ sperm per mini straw (according to the center operative procedures). Semen straws were filled, printed, and sealed before minimum chilling time, as per manufacturer guidelines (IMV Technologies, L’Aigle, France). After an equilibration time at 4°c for 4 hours, the straws were gradually cooled from 4 to -140°C in DigitCool programmable automatic freezer for 7 minutes (IMV Technologies, L’Aigle, France) and then submerged and stored in liquid nitrogen at -196^o^C until use. Several straws per sample were transported to the laboratory of IMV Technologies (L’Aigle, France) for post-thaw sperm evaluation.

### Post thaw semen analysis before CASA settings evaluation

Post-thaw motility was assessed using IVOS II(Animal Breeder, software version: v1.11.5) equipped with 10× negative phase contrast objective (Zeiss 10x NH IVOS-II 160nm). For analysis, two straws per batch were thawed at 37 °C for 30 s, emptied using a piston, diluted 1:4 (v/v) in prewarmed EasyBuffer B (IMV Technologies, L’Aigle, France) and incubated for 10 minutes at 37 °C. Then, 3 µL of diluted semen was loaded into a pre-warmed analysis chamber with a depth of 20 µm (Leja, Nieuw-Vennep, The Netherlands). For each sample, 8 fields were recorded and 588 to 1321 cells per sample were analyzed. Parameters measured included: 1) total motility (%); 2) progressive motility (%); 3) morphological abnormalities: bent tail, coiled tail, proximal droplet, distal droplet and distal mid-piece reflex (DMR); 4) concentration (10^6^ cells/mL); 5) kinematics parameters: amplitude of lateral head displacement (ALH, µm), curvilinear velocity (VCL, µm/s), straight-line velocity (VSL, µm/s), average path velocity (VAP, µm/s), linearity (LIN=VSL/VCL, %), wobble (WOB=VAP/VCL, %) and straightness (STR=VSL/VAP, %).

**Figure 1:**
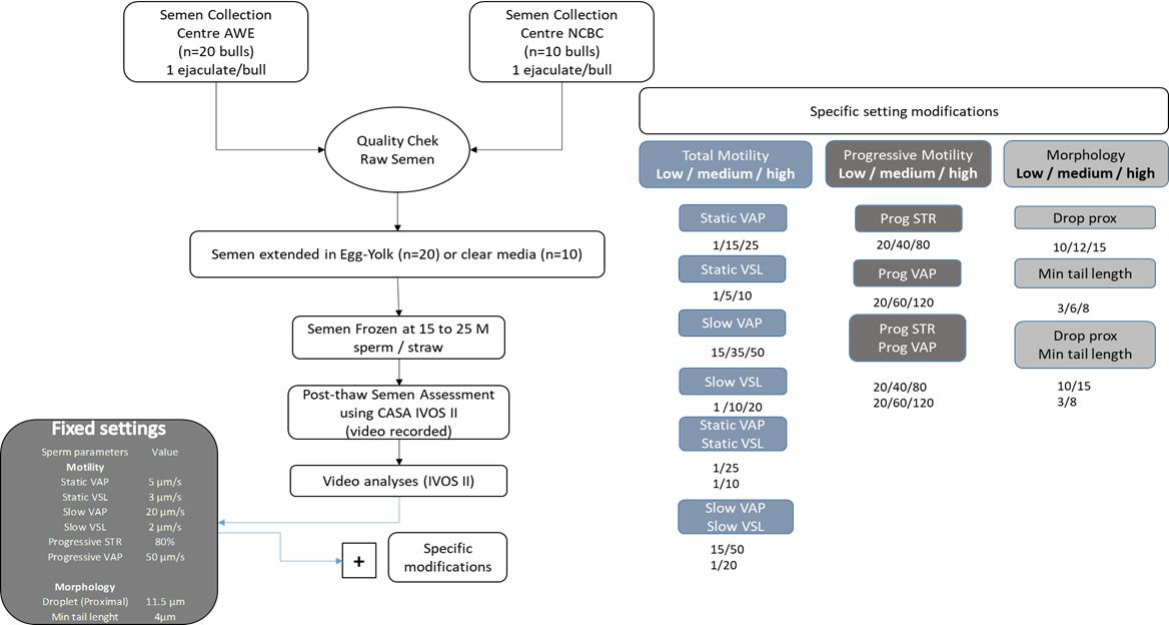
Experimental layout for fixed and adjusted settings on the IVOS II CASA. Ejaculates (n=30) were collected and processed by AWE and NCBC. Of which twenty were extended in egg yolk and the remainder in clear media. Classical IVOS II settings were applied to all video captures (Fixed settings) with additional modified settings for Low, Medium, and High threshold values alone or in combination for 1) total motility parameters: static and slow average velocity pathway (VAP; µm/s), straightness velocity (VSL; µm/s); 2) progressive motility parameters: progressive STR (%) and VAP (µm/s) and 3) morphology: proximal droplet (µm) and minimum tail length (µm).

All sperm analyses were captured on one IVOS II machine and each semen straw assessment was saved as a video recording. These videos (n=30) were then re-analyzed applying the following (Experimental design) different settings using several sets of values (Figure 1). This was done on five different machines.

### Experimental design

The fixed instrument settings are depicted in Figure 1 and were as follows:

1. image capture: frames per second = 60; number of frames = 30; illumination = red LED; photometer between 60 and 70. The light intensity may result in minor differences between different IVOS II units and samples analyzed (type of extender or slide). The photometer value is artificially created between 0-100 corresponding to the illumination intensity absolute values. Thus, the fixed photometer range allows the standardization of illumination intensity values. The *cell size* was between 6 and 70 µm^2^ and head minimum brightness was a minimum intensity of = 180;
2. Fixed motility settings were as follows: *static VAP* = 5 µm/s; *static VSL* = 3 µm/s; *slow VAP* = 20 µm/s; *slow VSL* = 2 µm/s; *progressive STR* = 80; *progressive* = 50;
3. Fixed Morphology settings were as follows: *droplet proximal head length* = 11.5 µm and *minimum tail length* = 4 µm.
4. Modified settings were as follows: Twelve different settings were created through the value modifications of both motility and morphology parameters. For each set, 2 or 3 different values ranging from Low, Medium, and High were tested. These settings are listed in the experimental flow chart (Figure 1). Samples (n=30, from saved videos) were re-analyzed under each modified setting value or a combination of values, leading to 1080 IVOS II analyses.

### IVOS II unit Standardized settings validation

Ten independent ejaculates of Belgian Blues bulls (AWE) diluted in egg yolk extender were used to validate the “standardized setting”. This setting was chosen according to the analysis of previous pictures of captured sperm cells. Videos were recorded for each of them, sent to all members, and then analyzed with settings currently used in semen production labs, and these results were compared with the same “standardized settings” across the units.

### Statistical evaluation

Comparisons among tested settings were done for the main IVOS II parameters, to highlight the “standardized setting”. General linear model analysis of SAS was used (SAS version 9.4, SAS Institute, Cary, NC, USA) to determine if there were differences among mean values of the different settings. If differences were detected among methods, Scheffe posthoc tests were used to determine the pairwise directional differences. Differences were considered significant when p ≤0.05. The variability of IVOS II results obtained through the “standardized settings” and the classical settings used in each lab, and among all the centers were analyzed to validate the “standardized settings”.

### Ethics approval and consent to participate

Not applicable since animals were not produced for experimental purposes. Indeed, the biological material was collected in semen production center for commercial bulls belonging to the breeding company AWE and NCBC.

## Results

The study showed the impact of different IVOS II settings on many semen parameters but only those relevant for semen quality evaluations are presented. All other data are listed in the Supplementary Material Table S1.

### Motility

#### Static or slow parameters

We analyzed IVOS II settings for *static* or *slow* VAP and VSL (with adjustments of specific modifications to Low, Medium, and High values) to examine the effect on the percentage of total motile cells and their kinematic parameters in extender with or without egg yolk. We found that increasing the static or slow parameters from Low to High values neither had a significant effect on total motility percentage nor their kinetic parameters, regardless of the considered extender (P>0.05).

#### Progressive motility parameters

When modified threshold values for STR progressive (%) were assessed, we found that increasing the range values from Low to High leads to a significant decrease in the percentage of progressive cells detected with IVOS II (Figure 22D). Indeed, in egg yolk extender, the percentage STR significantly decreased from 43.9±14.3 and 42.3±13.5 to 28.9±9.6%, respectively for Low, Medium, and High cut-off values (p<0.05). The same significant decrease was observed in clear extender for Low, Medium, and High cut-off values (46.3±8.4, 43.2±7.7, and 25.6±7.2%, respectively; p<0.05).

**Figure 2:**
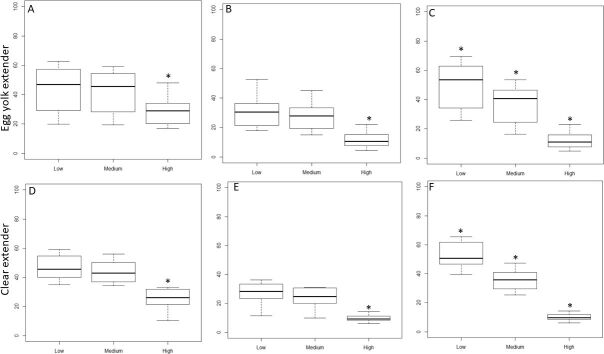
The effect of Low, Medium, and High modified settings on STR (A and D), VAP (B and E), and STR combined with VAP (C and F) on progressive sperm cell values from CASA in extender with egg yolk (A), (B) and (C) versus clear extender without egg yolk (D), (E) and (F). Asterix (*) represents statistical differences among range values (p < 0.05).

Similarly, the set threshold level for VAP progressive (%) had a large effect on the percentage of progressive cells (Figure 22E). Once again, both extender types presented the same significant changes when cut-off values moved from the Low, Medium to High (30.6±10.2, 27.6±9.4, and 11.6±5.2% for egg yolk extender and from 27.7±7.6, 24.2±7.0, and 9.7±2.4% for clear extender, respectively).

When both STR and VAP progressive (%) were adjusted the difference between Low, Medium and High value settings was even more pronounced for both extender types (p<0.05; Figure 22F). The percentage of progressive sperm decreased for Low, Medium, and High cut-off values from 49.5±15.2, 37.2±11.9 to 11.9±5.3% for egg yolk based extender and from 51.9±9.1, 35.8±7.3 to 10.0±2.4% in clear extender, respectively.

**Figure 3:**
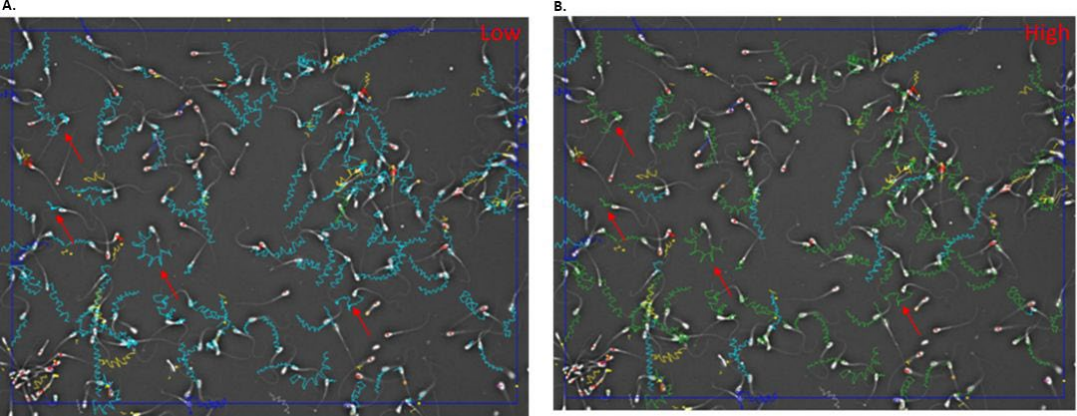
The effect of Low (A) versus High (B) value settings on STR and VAP progressive (%) in a clear extender. Blue trajectories represent progressive motile sperm cells, while green trajectories represent motile sperm cells. A huge majority of sperm cells have been declared as progressive cells with the Low threshold value. An important part of them is false positives, like those highlighted by red arrows. With the High threshold value, almost all the sperm cells have been classified from progressive cells into motile cells.

Scheffe analyses highlighted significant differences between the High value versus both Medium and Low values, except where both parameters are combined (Figure 22F) where all the three value ranges differed significantly. The picture (Figure 3) of captured sperm cells highlights the large majority of sperm cells colored blue as progressive cells and these are associated with the Low setting value (~70% of the sperm present in the picture). There are false positives, highlighted by red arrows. At High settings in the IVOSII almost all the sperm cells have been declassified from progressive cells into total motile cells. Indeed, only 11% of the sperm identified in these pictures are still progressive.

### Concentration

**Figure 4:**
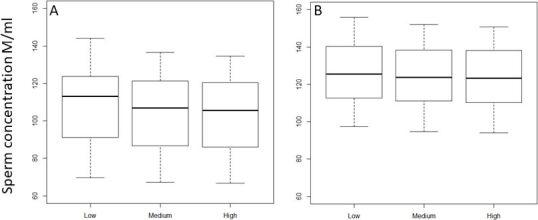
The effect of minimum tail length cut-off value modifications (ranging from settings of 3µm, 6µm to 8µm for Low, Medium to High values, respectively) on sperm concentration assessment in extender with (A) or without egg yolk (B). Asterix (*) represents statistical differences among range values (p<0.05).

When altering IVOS II settings, we also examined the effect of these modifications on sperm cell concentration (million/mL). Our results showed that there was no significant effect on concentration, regardless of which parameter value was applied and what extender was considered (Figure 4). However, when modifications were applied to *minimum tail length* parameters (ranging from 3µm, 6µm to 8µm for Low, Medium to High values, respectively), it resulted in minor but not significant fluctuations in the total sperm cells counted (between 2 to 4% according to the range values; P>0.05; Figure 4).

### Morphological abnormalities

Within IVOS II setting ranges, the following morphological parameters were adjusted to examine the effect on mean counts (%) of sperm cells detected by the CASA system: *droplet proximal head*, *minimal tail length*, and a combination of these two parameters.

**Figure 5:**
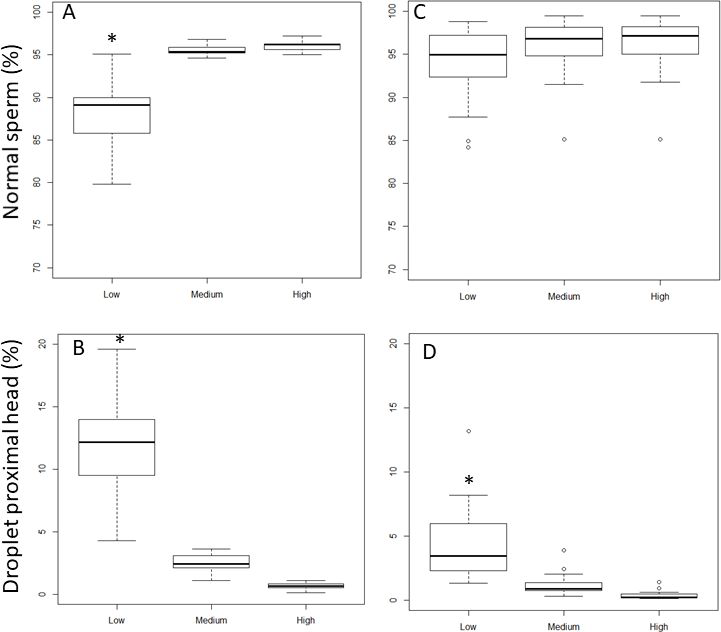
The effect of Low (10µm), Medium (12µm) and High (15µm) setting modifications of *droplet proximal head length* on normal sperm cell (A and C) and droplet proximal head percentages (B and D) in clear (A and B) or in egg-yolk (C and D) extender. Asterix (*) represents statistical differences among range values (p<0.05).

The increase of *droplet proximal head length* setting value from 10 (Low), 12µm (Medium) to 15 µm (High, Figure 5A) significantly increased the percentage of sperm cells identified as normal for clear extender (88.0± 4.7% to 95.0 ± 0.6% and 96.0 ± 0.6% for Low to Medium and High respectively; P<0.05). In egg-yolk-based extender, the percentage of normal cells did not increase significantly (94.0 ± 4.2% to 96.0 ± 3.8% and 96.0 ± 3.7% for Low, Medium, and High, respectively; P>0.05, Figure 5B). The percentage of detected proximal droplets decreased significantly with increasing settings values from 10 µm (Low), 12 µm (Medium) to 15 µm (High) to yield results of 12.2 ± 4.7%, 2.5 ± 2.7% to 0.6 ± 0.2%, respectively, when samples were analyzed in clear extender (Figure 5C). This difference was also observed in egg-yolk-based extender where the percentage of detected proximal droplet heads decreased significantly from 4.4 ± 2.8% for Low threshold value, to 1.2 ± 0.8% and 0.3 ± 0.3% (for Medium and High threshold values, respectively; p<0.05). The percentages of DMR were not affected by the *droplet proximal head length* cut-off value modifications, for both extender types.

An increase of the *minimum tail length* threshold value from 3 µm, 6 µm to 8 µm (Low, Medium and High settings respectively) has no significant effect on the percentage of normal cells and cells with proximal droplets detected by the IVOS II, nor is it affected by extender type. However, under the same conditions, a significant decrease in the percentage of detected cells with DMR from 2.6 ± 0.4%, 1.5 ± 0.4%, and 1.4 ± 0.3% (p< 0.05) was observed in clear extender, but not in egg yolk extender (3.6 ± 2.0%, 2.9 ± 1.8%, and 2.5 ± 1.7% respectively for Low, Medium and High values).

### Validation of the “standardized settings” across different IVOSII units

**Figure 6:**
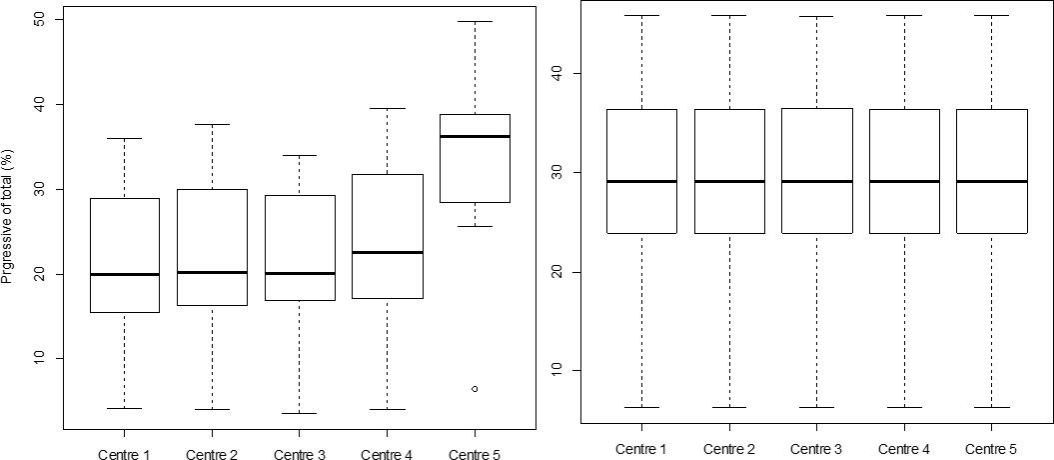
The effect of the IVOS II settings on the identification of progressive sperm percentages. The motility of 10 Belgian blue bulls was assessed by using an IVOS II set up with center selected settings (A) and “standardized settings” the same across all centers (B). Asterix (*) represents statistical differences among range values (p<0.05).

The results of sperm characteristics obtained when looking at pre-existing settings in use across different centers using IVOS II, resulted in a range of variability between 1.7% (VCL) to 31.2% (DMR) depending on the evaluated IVOS II parameter (20.2%, 3.6%, 3.8%, 2.1%, and 21.4% respectively for distal droplets, normal sperm, ALH, motile and progressive sperm). There was no variation obtained when the “standardized settings” were applied to each IVOSII machine located at each AI center involved in the study. The boxplots for the percentage of progressive sperm cells obtained for each center with (Figure 6A) or without the “standardized settings” (Figure 6B), clearly illustrate the impact of the applied settings on the variability among IVOSII units within centers. The hierarchy among analyzed bulls with both settings is also conserved within each center (R between 0.97 and 0.98 according to the center).

### The “standardized settings” for IVOSII units

Based on the results of this study, the “standardized settings of the IVOSII CASA system are the following: 1) proximal droplets at 12 µm; 2) minimum tail length at 4 µm; 3) *STR* at 60%; 4) *VAP progressive* of 40 µm/s; 5) static and slow VSL at 3 µm/s, 2 µm/s, respectively; and 6) static and slow VAP at 5 µm/s and 20 µm/s, respectively.

## Discussion

Several studies drove comparisons among different settings pre-established for different species, or among different slide types, or frame rates, but to the best of our knowledge, the present study is the first to compare the effect of altering CASA settings on values obtained for several different semen quality parameters for bovine. The aim of the present study was to evaluate whether altering settings of IVOS II would change obtained results. In this study, it was clear that when setting threshold values were changed, different results were achieved, in particular for progressive motility. A change from Low to High cut-off values resulted in 20%, and 40% difference in results obtained for sperm straightness and average path velocity.

With the increased global demand for bovine semen, the information exchange between AI centers including the fertilization capacity of the dose (male fertility, non-return rates) as well as quality parameters evaluated in the lab (total and progressive motility, total live cells per ejaculate, etc.) are becoming a standard requirement for routine semen processing at AI centers. Due to the difference in sample processing, applied protocols, and data handling, such information may become unreliable and difficult to interpret. It was the intention of this study and contribution from each center to address common issues with the interpretation of results from CASA.

Assessment of ejaculate quality is primarily based on post-thaw semen evaluation using quality parameters such as sperm motility, morphology, viability, biochemical estimations of enzyme release, membrane, and acrosome integrity (Kathiravan et al., 2011; Nagy et al., 2003). Among these, motility is the most commonly used method of assessment and previous studies have even found relationships between motility and field fertility (Januskauskas et al., 2003). However, motility scores between AI centers are not always comparable as protocols for sample freezing, thawing, preparation, loading, slides, and hardware differ between centers. Computer-assisted semen analysis facilitates objective classification of a sperm population based on motility (Hoflack et al., 2007; Broekhuijse et al., 2011). Indeed, this technology was developed to replace the subjective assessment of sperm motility, to reduce differences between technicians and experience level. However, in this study, it was clear that different settings adjustments do lead to different final analysis results and this is similar to previous findings (Simonik et al., 2015). The AI centers involved in this study conducted preliminary trials to look at variables that can affect results from CASA (unpublished data) and their results were very similar to those found previously, where external factors like sample temperature (Birks et al., 1994), sample dilution factor (Farrell et al., 1996), mixing, pipetting, time to analysis, technician and the use of counting chambers (Bailey et al., 2007) caused a large degree of variation or loss of cell motility. In a routine environment, both Viking Genetics and CRV reported technical variability among repetitions of the same sample or between technicians, in the order of 6% (unpublished data).

Surprisingly there was no significant impact of either setting threshold values or extender type (egg yolk versus clear extender) on the total motile percentage of spermatozoa when either slow or static parameters were adjusted. It could lead to questioning as to whether chosen settings were extreme enough to detect any differences? Tested range values were selected to be consistent with the value variations generally observed in published works (Chaveiro et al., 2006; İnanç et al., 2018; Lenz et al., 2011). Some kinematic parameters (such as VSL) increased with the threshold value modifications, but this was not significant. Indeed, a fraction of the sperm population classified as slower cells within CASA was considered as non-motile cells and automatically it enhanced the kinematic means of the motile population. Progressive sperm cells could be defined as the faster cells with a linear trajectory. STR progressive (%) and VAP-progressive (µm/s) are the IVOS II parameters that define this sperm population. The progressive percentage is an important indicator of semen quality, widely used worldwide. Therefore, a good, reliable, and comparable method of evaluation for this parameter is required. However, the results from this study highlight that the IVOS II settings largely influence the percentage of progressively motile spermatozoa detected. Indeed, Higher cut-off values for straightness and/or velocity lead to some progressive cells being classified as motile but not progressive. Lowering the cut-off values leads to classifying a lot of motile sperm cells as progressive, and thus altering the correct numbers in both populations.

As expected, total sperm cell concentration was not significantly impacted despite the setting used for both extender types. All the applied modifications had classified or declassified sperm cells in different subcategories. However, despite the subcategories, each considered sperm cell was counted. The only important reported difference existed between the two extender types. Debris and particles within egg-yolk extender can obscure the evaluation of concentration, but it was impossible to evaluate the effect of an individual bull or the collection center since ejaculates were not split samples.

Spermatozoa morphology is a fundamental element affecting its ability to reach the site of fertilization in the female tract, fertilize an ovum, and initiate embryogenesis (Saacke, 2008). Proximal droplets can be identified as a regularly shaped remnant of cytoplasm under the plasma membrane in the neck region of the spermatozoa. High percentages of proximal droplets can be found in the semen of prepubertal bulls, while in mature bulls, they are considered a sign of abnormality in spermiogenesis or epididymal sperm maturation (Amann et al., 2000; Carreira et al., 2012; Lasiene et al., 2013). Whatever the origin of proximal droplet formation, that abnormality impacts the spermatozoa quality traits, both *in vivo* (Al-Makhzoomi et al., 2008) and *in vitro* (Carreira et al., 2012). The present study found that different droplet proximal head length thresholds had an impact on the percentage of detected proximal droplets and, as a consequence, on the percentage of morphologically normal spermatozoa calculated. These results highlight the need for manufacturers to ensure that settings are validated and possibly set into the system rather than users defining such settings and achieving large variability in morphology results.

In clear extender, the percentage of normal cells differed significantly when the Low threshold value was applied, mainly due to the increase of proximal droplets (~x5). It is not the case with egg-yolk extender, where proximal droplets were also increased but at a lower rate (~x2).

IVOS II detects the sperm cells according to the contrast (brightness) of sperm cells to the background, sperm head area, and elongation. The transparency of the extender affects the sperm contrast and head brightness and needs to be adjusted to correctly identify sperm cells. The incorrect configuration will lead to misidentification of sperm cells and over/underestimation of normal sperm population. Too Low illumination or too High *minimum head brightness* may lead to omitting some sperm cells, whereas too High illumination or too Low *minimum head brightness* will incorrectly overscore the number of proximal droplets in the cells.

Although it should be expected to obtain the same results for motility parameters across different members CASA Units, in this study (for the same 10 Belgian Blue ejaculates) this validation step was a required step to ensure that the new raw values were not lower than those obtained with the home-made settings of each center. The results obtained from different IVOSII units highlighted a reduction in the variability among AI centers (with the standardized settings) and on average a higher percentage of progressive cells in 4 of the 5 AI centers participating. The bull classification according to their quality remains the same when settings standardized settings are applied.

Based on the findings of this study, we would suggest that to successfully compare results and determine if samples have deteriorated in quality (*i.e.,* during transit or handling, etc.) or are suitable for use in the field we would suggest that other AI centers would also consider the use of the “standardized settings” (Table 1 and Supplementary Material). This work was not designed to provide an international agreement on minimum semen quality acceptance thresholds, but it is the first step towards consensus on settings within one CASA platform and is suggestive that other CASA units may need the same level of optimization which could lead to further towards general guidelines and internationally accepted standards.

**Table 1:** Suggested setting values for “standardized settings” as per bull frozen semen Standardized QualiVets settings on the IVOSII CASA machine.

**Setup**	**Name**	**Bull frozen semen Standardized QualiVets settings**
Analysis limits	Min motility Percent	0
	Min progressive Percent	0
	Min total Count	200
Calibration	Objective	1:Zeiss 10x NH IVOS-II 160nm
	Objective Magnification X	1.2
	Objective Magnification Y	1.2
Camera	Exposure (Ms)	16
	Gain	300
	Integrate Enabled	FALSE
	Integrate Time (Ms)	500
Cell Detection	Elongation Max (%)	90
	Elongation Min (%)	1
	Enable Advanced Tail Detection	FALSE
	Head Brightness Min	168
	Head size Max (µm^2^)	70
	Head size min (µm^2^)	6
	Static Tail Filter	TRUE
	Tail Brightness Min	96
	Tail Min Brightness Auto Offset	8
	Tail Min Brightness Mode	AUTO FIRST FRAME
Chamber	Capillary Correction	1.3
	Chamber Depth (µm)	20
	Chamber type	CAPILLARY
Illumination	Contains Auto illum Calibration	TRUE
	Histogram Smooth Width	0
	Illumination Primary	LED
	Max Photometer	70
	Min Photometer	60
Kinematics	Cell Travel Max (µm)	15
	Enable Motile Static Collision Avoidance	FALSE
	Motile cells require a tail	FALSE
	Motile require Tails Max VSL (µm/s)	0
	Progressive STR (%)	60
	Progressive VAP (µm/s)	40
	Slow VAP (µm/s)	20
	Slow VSL (µm/s)	2
	Static Algorithm	LENGTH
	Static VAP (µm/s)	5
	Static VSL (µm/s)	3
	Static Width Multiplier	0.5
Morph		
	DMR Droplet to tail end Max (µm)	5
	DMR Tail Length Max (µm)	15
	Droplet Distal Distance Min (µm)	4
	Droplet Proximal Head Length (µm)	12
	Min Tail Length (µm)	4
	Morph Normal Minimum Percentage	0
	Tail Bend Angle Averaging Length (µm)	5
	Tail Bending Angle Rate Min (°/µm)	20
	Tail coiled Angle Min (°)	180
Stage	Stage Temp	37
Viadent	Viadent Fluo Sperm	NON VIABLE
Video Capture	Frame Capture Speed (Hz)	60
	Frame count	30

## Conclusions

High precision and the use of standardized settings of CASA system are essential for reaching guaranteed levels of insemination dose quality, efficient semen dose production, and field fertility results. Our current studies confirmed concerns on the variability of results achieved using different CASA settings and would urge centers to validate systems and ideally engage in a standard-setting for the CASA IVOS II system. If applied, this is an important step in the standardization of semen quality assessment in the bovine AI industry. Next studies will focus on combining these settings with a standard operating procedure (similar to the WHO standard in the human industry). This will bring us even closer to worldwide knowledge and uniform semen processing and assessment. The standardized settings, named “Bull frozen semen Standardized QualiVets settings” are proposed in Table 1 and supplementary data.

## Supplementary Material

Supplementary material accompanies this paper.

Table S1 General database of the experiment

Supplementary File 1 IVOS II setting file

This material is available as part of the online article from https://www.scielo.br/j/ar
